# Supplementary material for: Advanced CMOS manufacturing of superconducting qubits on 300 mm wafers
Source: Nature. 2024 Sep 18;634(8032):74–9. doi: 10.1038/s41586-024-07941-9 (PMC11446867; doi:10.1038/s41586-024-07941-9)
Supplement: Supplementary file 1 — Supplementary Sections I–VI, Figs. 1–8 and Table 1. [file 41586_2024_7941_MOESM1_ESM.pdf]

nature portfolio

https://doi.org/10.1038/s41586-024-07941-9

Supplementary information

Advanced CMOS manufacturing of superconducting qubits on 300 mm wafers

In the format provided by the
authors and unedited

Supplemental material
Advanced CMOS manufacturing of superconducting qubits on 300 mm wafers

J. Van Damme[1,2], S. Massar[1], R. Acharya[1], Ts. Ivanov[1], D. Perez Lozano[1], Y. Canvel[1], M. Demarets[1,2], D. Vangoidsenhoven[1], Y. Hermans[1], J.G. Lai[1], A. M. Vadiraj[1], M. Mongillo[1], D. Wan[1], J. De Boeck[1,2], A. Potočnik[1*], K. De Greve[1,2]

[1]Imec, Kapeldreef 75, Leuven 3000, Belgium
[2]Department of Electrical Engineering (ESAT), KU Leuven, Leuven 3000, Belgium

I. Device designs

The mask design of one die on the wafer in this work constitutes 20 different sub-dies of mostly fixed frequency qubits coupled to read-out resonators and a common feedline. A large parameter space is covered with these designs to accommodate the a priori unknown values of co-planar waveguide (CPW) phase velocities, JJ barrier resistivity and thickness of the new process. Two sub-die designs D1 and D2 were selected for this study, with their optical micrographs shown in Figure S1 and their design parameters summarized by Supplementary Table I. Actual qubit parameters of all measured qubits can be found in a public data repository referenced in the data availability statement.

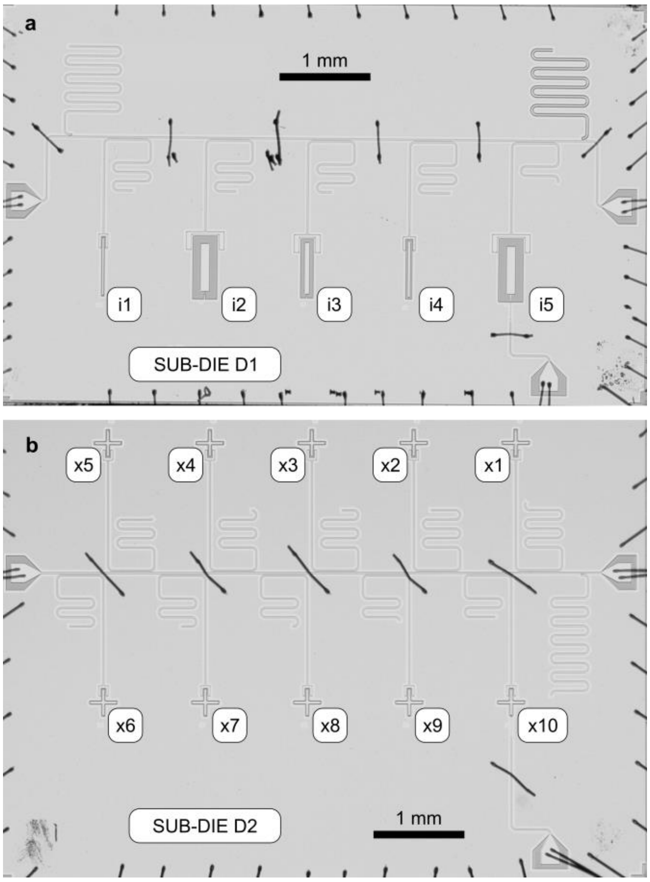

SUPPLEMENTARY FIG. 1. **a**, Optical micrograph of an example sub-die D1, wire-bonded into an aluminum measurement package. **b**, Optical micrograph of an example sub-die D2, wire-bonded into an aluminum measurement package.

SUPPLEMENTARY TABLE I. **Qubit design parameters.** The qubits on sub-dies D1 and D2 as labelled in Supplementary Figure 1 were designed with the parameters summarized in this table. The planar capacitor width and gap to the ground plane, the nominal critical junction dimension (cd), the CPW read-out resonator frequency (f_r), the qubit frequency (f_{qb}), the charging energy of the transmon (E_C), the Josephson energy of the transmon (E_J), the coupling strength between qubit and read-out resonator (g) and if a symmetric superconducting quantum interference device (SQUID) is used to make the qubit flux tunable.

Name	width (μm)	gap (μm)	cd (nm)	f_r (GHz)	f_{qb} (GHz)	$\frac{E_C}{h}$ (MHz)	$\frac{E_J}{h}$ (GHz)	g (MHz)	SQUID
i1	13	13	120	7.2	3.94	203	10.56	77.7	no
i2	90	90	90	6.3	2.90	202	5.94	55.0	no
i3	48	48	100	6.6	3.27	206	7.33	68.8	no
i4	24	24	110	6.9	3.59	203	8.87	68.0	no
i5	90	90	110	7.5	5.15	202	17.75	85.6	yes
x1	20	13	119	6.0	3.08	231	5.94	52.5	no
x2	20	13	131	6.2	3.45	231	7.33	57.1	no
x3	20	13	143	6.4	3.82	231	8.87	61.8	no
x4	20	13	156	6.6	4.19	231	10.56	66.5	no
x5	20	13	168	6.8	4.48	231	12.02	70.7	no
x6	20	13	180	7.0	4.74	231	13.37	74.7	no
x7	20	13	191	7.2	5.11	231	15.42	79.6	no
x8	20	13	205	7.4	5.47	231	17.62	84.5	no
x9	20	13	220	7.6	5.77	231	19.48	88.9	no
x10	20	13	165	7.8	6.07	231	21.47	93.4	yes

II. Josephson junction normal resistance wafer location dependence

A clear centre-to-edge variation is observed in the qubit frequencies measured across the wafer (main text Figure 4c). This trend is shared with the normal resistance measurements performed on Josephson junction (JJ) test arrays across the wafer (Supplementary Figure 2d). Sampled inspections of JJ electrode dimensions across the wafer (Supplementary Figure 2a,b) reveal that some, of the location dependence results from etch non-uniformity between the centre and the edge of the wafer. The barrier oxidation process, on the other hand, does not show such a centre-to-edge dependence when replicated on un-patterned, blanket wafers, as illustrated by the resistance-area product wafer-map in Supplementary Figure 2c. This analysis clearly identifies the patterning process as a contributor to the centre-to-edge dependence of qubit frequencies measured across the wafer. However, other sources like bottom electrode morphology and roughness (determined by etch and argon milling), or aluminum grain size are likely to exhibit centre-to-edge variation as well. Large-scale inspections of device critical dimensions could be developed to quantify the contributions of each processing step to the centre-to-edge variation and improve the fabrication methods of this work towards higher wafer-uniformity. Note that our process includes a spurious junction at the overlap with the bottom electrode capacitor[1].

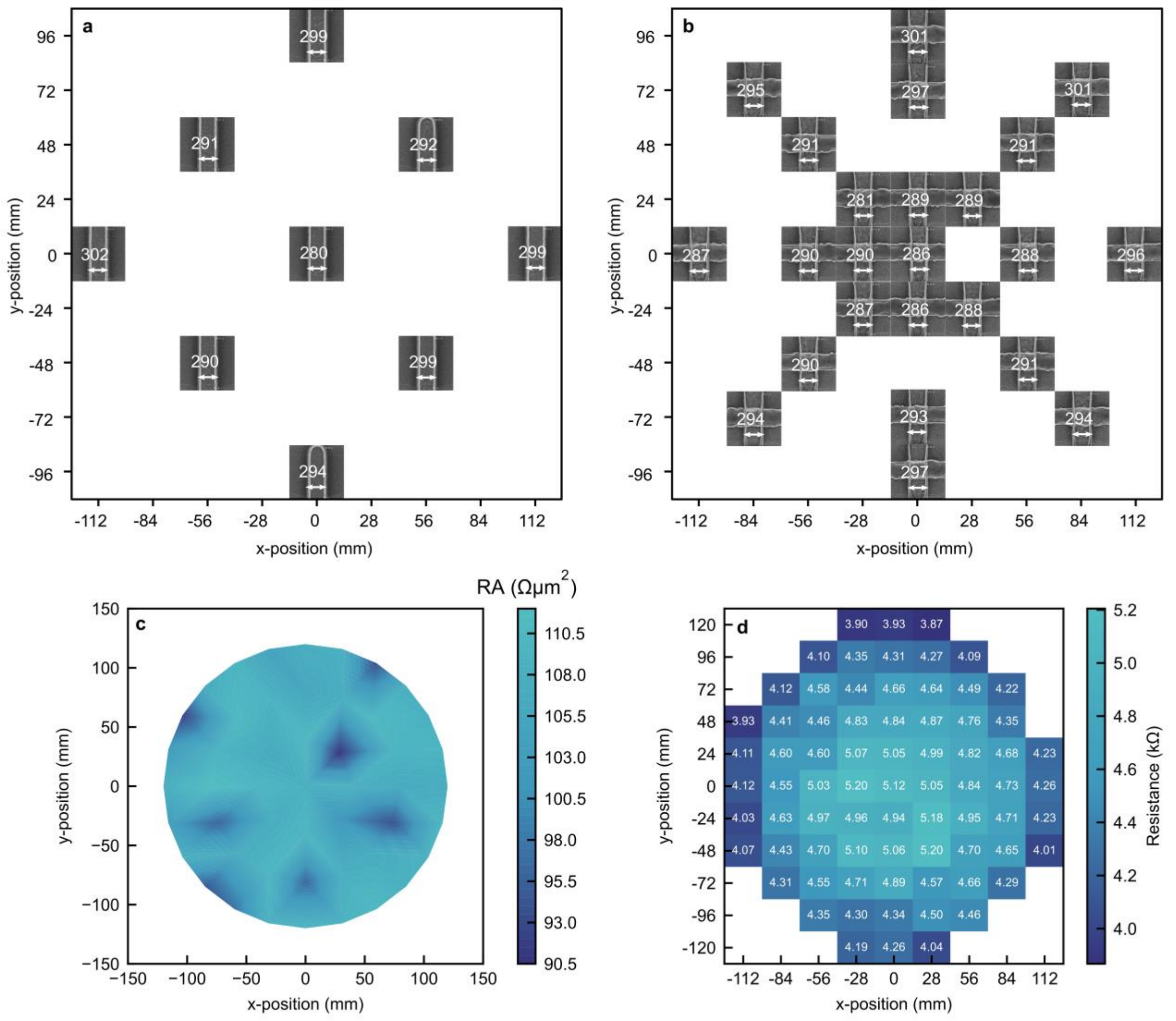

SUPPLEMENTARY FIG. 2 **JJ resistance variability across the wafer a**, Scanning electron microscope images of the bottom electrode of junctions across the wafer (designed width = 200 nm). The measured width of the electrode (in nm) is annotated. **b**, Scanning electron microscope images of the top electrode of junctions across the wafer (designed width = 200 nm). The measured width of the top electrode (in nm) is annotated. **c**, Resistance-area product extracted via current in-plane tunneling (CIPT) characterization on a wafer where the barrier oxidation process was replicated on blanket without patterning. **d**, Average normal resistance of Josephson junctions (8 copies per die), with designed electrode widths of 200 nm, measured across the wafer.

The reported scanning electron microscope (SEM) images in Supplementary Figure 2a,b were taken on the 300 mm wafer using a Hitachi CD-SEM tool with included dimension measurements. The SEM image of main text Figure 1c was taken with a HELIOS 1200AT tool. The Resistance-area product on the blanket wafer (Supplementary Figure 2c) were extracted using current in-plane tunneling (CIPT) on a Capres microHall-A300 (MH300) with probe L8pp.A03. The JJ test array normal resistances were measured at room temperature with a 300 mm wafer prober Tokyo Electron SYSTEM VI PRECIO tool. Additional JJ test-array normal resistance measurements were performed on diced coupons with a SUSS MicroTec iVista high-resolution wafer prober (data used for the single-die statistics of main text Figure 4a and aged data of Figure 4b,d).

III. Josephson junction energy dispersive X-ray analysis

The composition of the overlap Josephson junctions (JJ), fabricated with the process described in this work, is investigated with energy dispersive X-ray (EDS) analysis. A focused ion beam (FIB) specimen is extracted with a Helios 450, HP FIB lift-out system, and further analysed with a scanning transmission electron microscope (STEM) Titan G2 at 200 kV. Like in our previous work[1] traces of Si and Ar are detected inside the junction barrier, while no C is present.

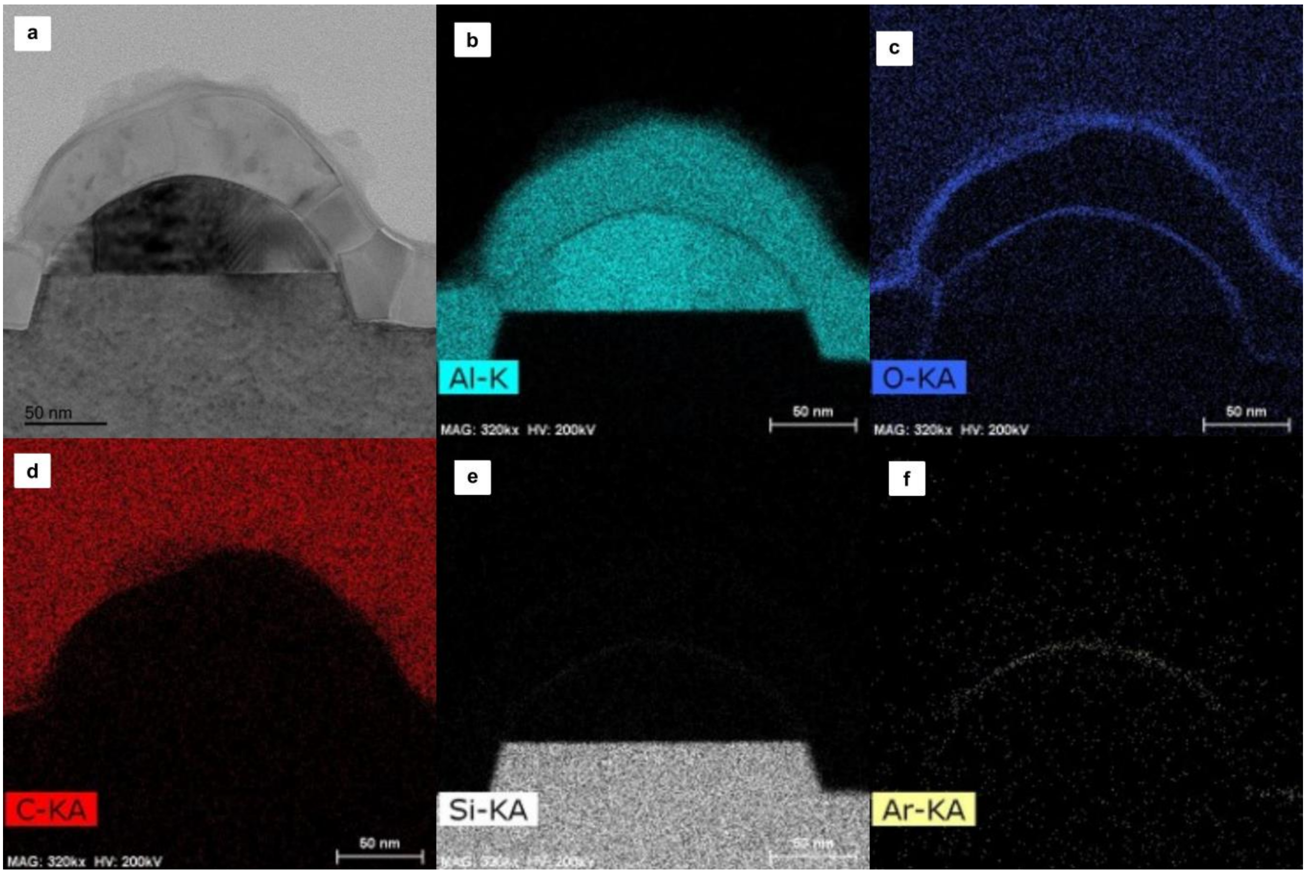

SUPPLEMENTARY FIG. 3. **a**, Scanning transmission electron microscopy (STEM) image of the cross-section of a JJ. **b**,**c**,**d**,**e**,**f** Energy dispersive X-ray (EDS) analysis of the chemical composition of the JJ cross-section. The detected atomic concentrations of Al, O, C, Si, and Ar are coloured respectively.

IV. Batch-to-batch reproducibility

The data presented in the main text is collected from one wafer (Batch 1, Wafer 1). In Supplementary Figure 4 we demonstrate batch-to-batch and within-batch reproducibility by measuring JJ normal resistance at room temperature across multiple wafers. We define a batch as a set of wafers that are processed sequentially at each step of the process, without major time interruption or, with identical tool settings. The observed variation in JJ resistances in the wafer-to-wafer and batch-to-batch comparisons could be due to variations in deposition, lithography, etch, argon milling, and barrier oxidation. The barrier oxidation uniformity and reproducibility is likely playing a dominant role, due to the exponential dependence of the tunnel barrier resistance on oxide thickness. A prolonged barrier oxidation to guarantee a more uniform saturated oxide thickness could help[2]. We note that currently our efforts prioritize process optimization in terms of qubit metrics, while more extensive wafer-to-wafer and batch-to-batch reproducibility studies, including cryogenic qubit measurements, are planned for future works.

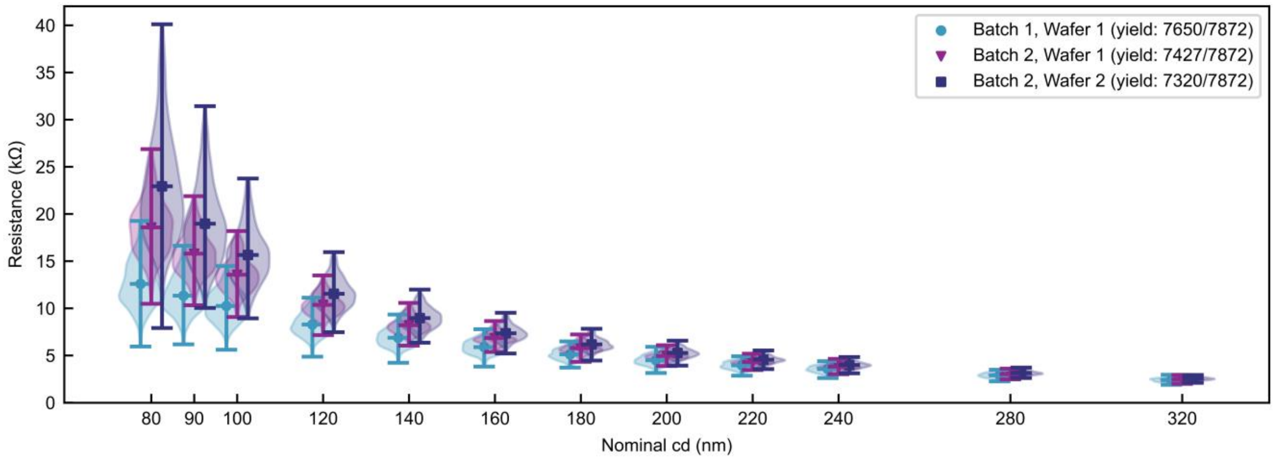

SUPPLEMENTARY FIG. 4 **Batch-to-batch and wafer-to-wafer reproducibility.** Josephson junction normal resistance values measured on 7872 test structures across each of the three wafers. The distribution of resistances at each designed junction lead critical dimension (cd) is filtered for outliers beyond 1.5 times the interquartile range. The filtered distributions are visualized with violin plots including the mean and extrema at each cd (offset included for improved visibility).

V. Scanning for avoided crossings in the qubit spectrum

Two-level systems inside the JJ barrier are expected to couple strongly to the qubit, typically resulting in an avoided crossing between the TLS and the qubit transition frequencies with the splitting on the order of 10 MHz[3,4]. Supplementary Figure 5 illustrates how we scanned the qubit spectrum of a flux tuneable qubit. Every Ramsey sequence of this experiment contains 1000 points, spaced with 2 ns, resulting in an FFT detection bandwidth of 250 MHz and a frequency resolution of 500 kHz (sensitive to avoided crossings of $2g > 500$ kHz). A total of 150 equally spaced flux pulse amplitudes was used to scan ~140 MHz of the qubit spectrum. No double frequencies or avoided crossings were observed within this scanning range.

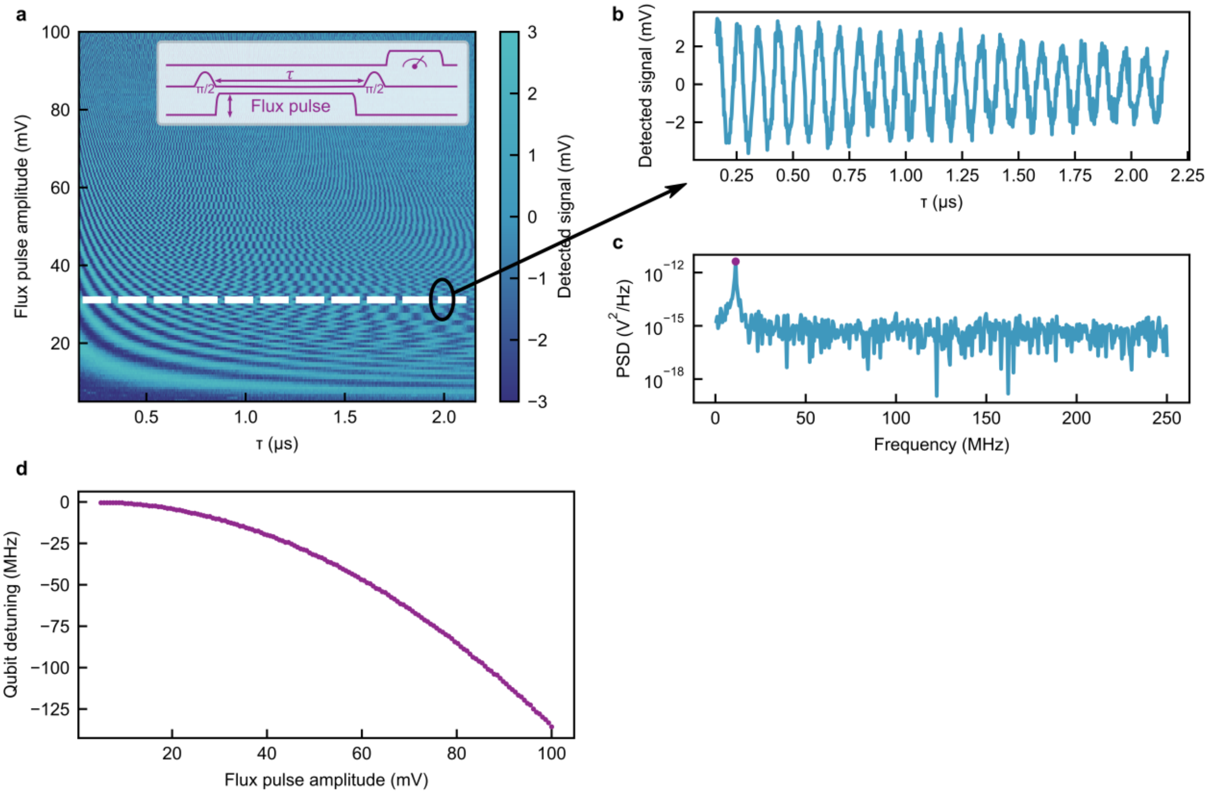

SUPPLEMENTARY FIG. 5 **Qubit frequency spectrum. a**, Detected readout signal as a function of Ramsey delay τ and flux pulse amplitude following the pulse sequence diagram of the inset. **b**, Ramsey oscillations at 30.5 mV applied flux

offset. **c**, Power spectral density of the data in **b**, with highlighted qubit frequency, extracted with a peak detection algorithm. **d**, Extracted qubit frequency detunings as a function of each applied flux pulse amplitude, showing no signs of avoided crossings.

VI. Coherence data of all measured qubits

All qubit time-averaged relaxation and coherence times measured across the wafer are summarised in Supplementary Figure 6. The cumulative distribution functions are separated for each capacitor geometry of the qubits in Supplementary Table I, and between fixed-frequency or frequency tuneable qubits (with SQUID). Most of the measured qubits have capacitor designs with intentionally large interface electric field participation ratios according to the design of experiment (DOE), compromising their relaxation times. Additionally, we note that the frequency tuneable qubits are Purcell-limited in their relaxation times.

In Supplementary Figures 7 and 8, the qubit quality-factors $Q = 2\pi f_{qb} \langle T_1 \rangle_t$ of each fixed frequency qubit are plotted at their respective die locations. In Supplementary Figure 8 the Q-factors of all nine fixed frequency qubits x1-x9 (see Table I) with identical capacitor geometries have been averaged on each die location. The qubits on sub-die D1 are separated by capacitor geometry in Supplementary Figure 7 with increasing interface participation ratio ordering. Four of the non-functional qubits (7 out of 400) were of design i2 (one was of design x7, and two were of design i5), suggesting that the smallest JJ area (cd of 90 nm) is at the edge of our process yield. The random frequencies of the TLS in our devices can result in unlucky defects resonant with the qubits, causing a dominant non-systematic loss channel, known by the community and not the target of this work. It is only when minimizing this random contribution of unlucky resonant TLS as much as possible (by taking the best performing devices at each location) that the more systematic underlying location dependence of the T_1 upper bound (Figure 2a) is clearly noticeable. The identification of the centre-to-edge dependent losses will be the subject of a future study with superconducting high Q-factor resonators.

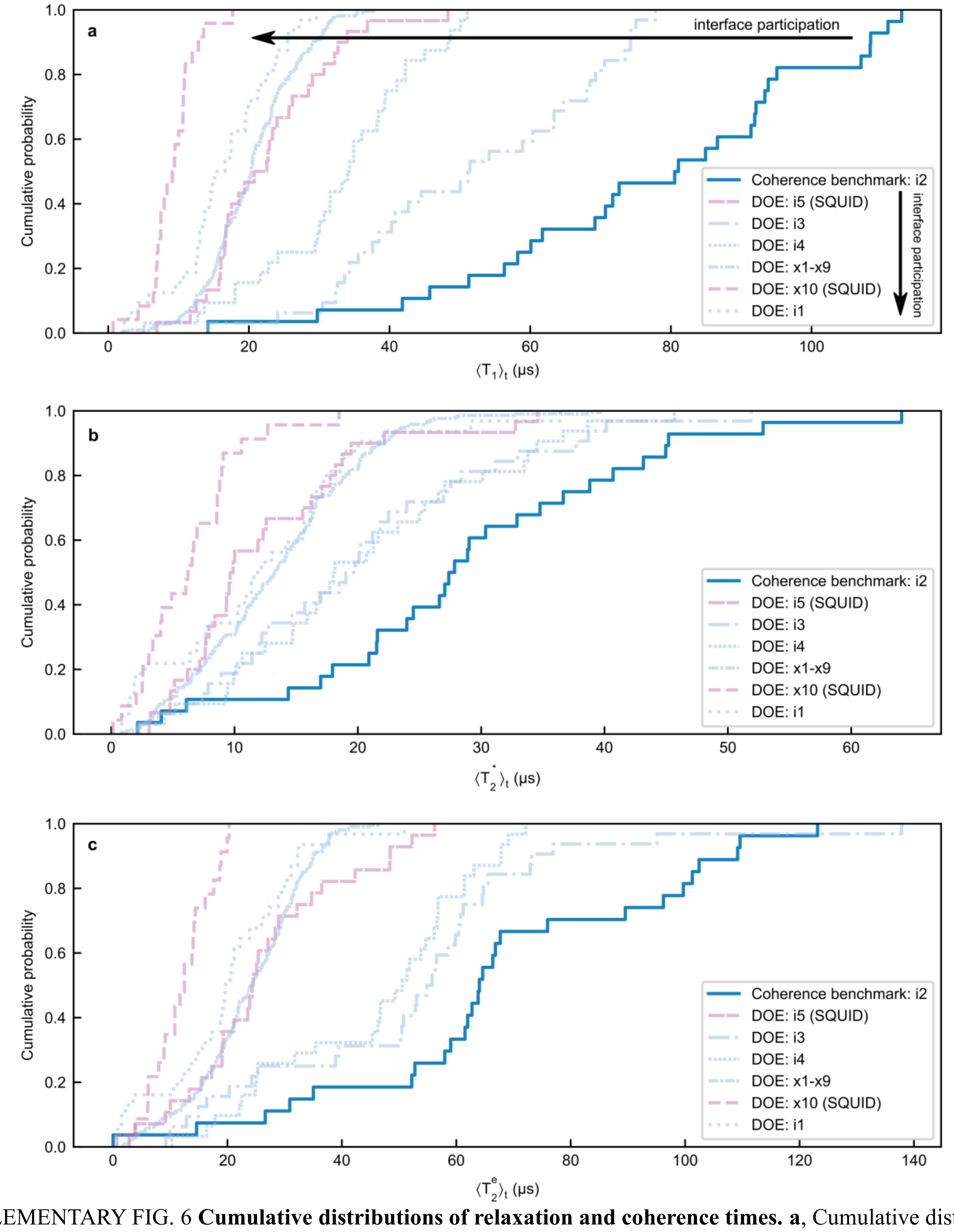

SUPPLEMENTARY FIG. 6 **Cumulative distributions of relaxation and coherence times. a**, Cumulative distribution function of the time averaged relaxation times $\langle T_1\rangle_t$ of all qubits measured in this work. **b**, Cumulative distribution function of the time averaged Ramsey decoherence times $\langle T_2^*\rangle_t$ of all qubits measured in this work. **c**, Cumulative distribution function of the time averaged Hahn echo decoherence times $\langle T_2^e\rangle_t$ of all qubits measured in this work. The distributions are separated between design of experiment (DOE) geometries with intentionally increased capacitor interface participation ratios and coherence benchmark designs as detailed in Supplementary Table I.

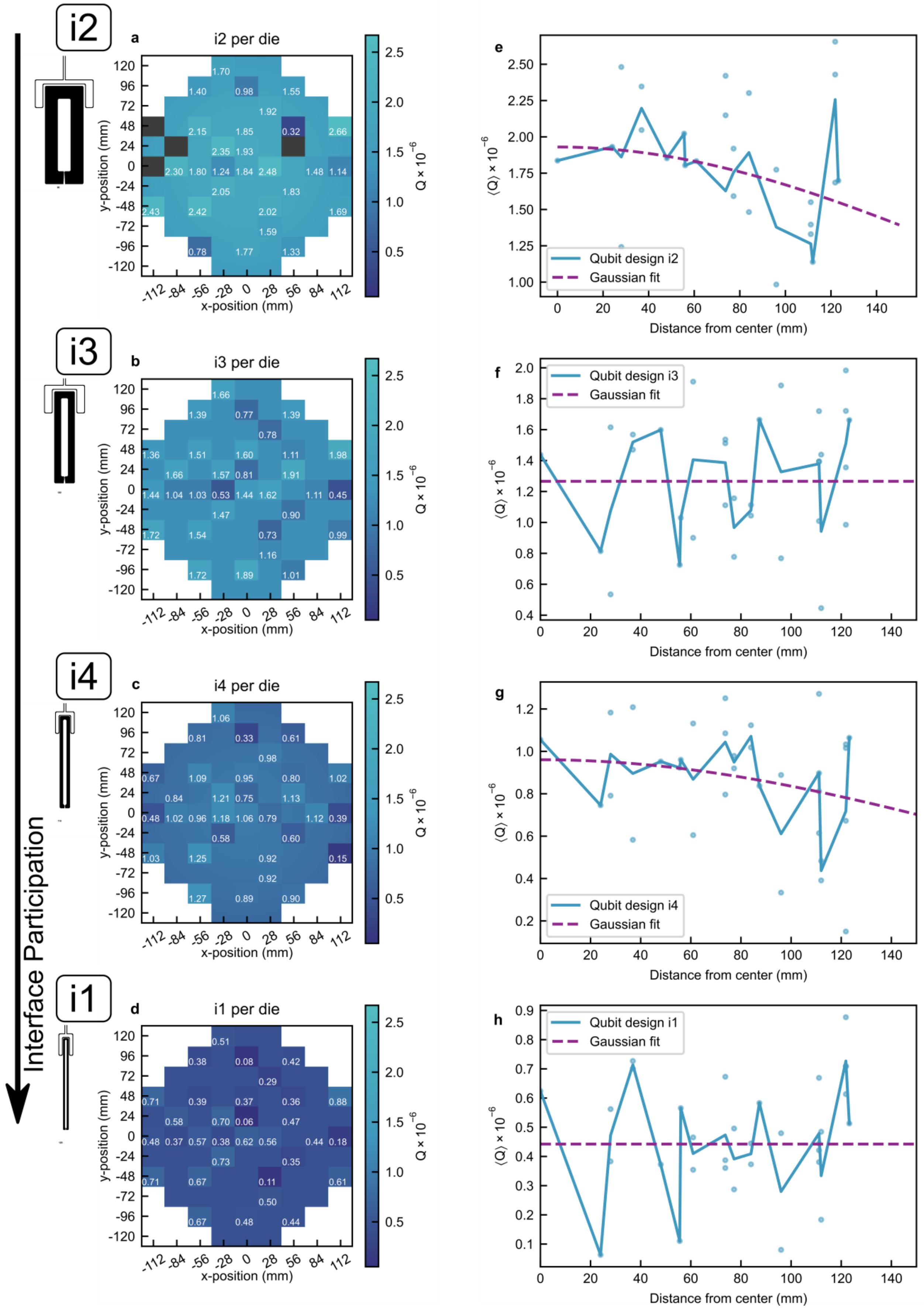

SUPPLEMENTARY FIG. 7 **Sub-die D1 Q-factor wafer maps.** Measured qubit quality factor $Q = 2\pi f_{qb}\langle T_1\rangle_t$ at each location across the wafer for each qubit design i2 (**a**), i3 (**b**), i4 (**c**), i1 (**d**) according to increasing capacitor interface

electric field participation (see Extended Data Figure 4). Blacked out locations correspond to non-functional qubits, whose JJ dimensions were at the limit of the process window. The background colour represents the Gaussian fit to the average value as a function of distance from centre. **e**,**f**,**g**,**h**, Q-factors plotted as function of distance from the centre of the wafer, the solid lines represent the average values, while the dashed lines are gaussian fits to the data, acting as guides to the eye and used as the background colour in **a**,**b**,**c**,**d**.

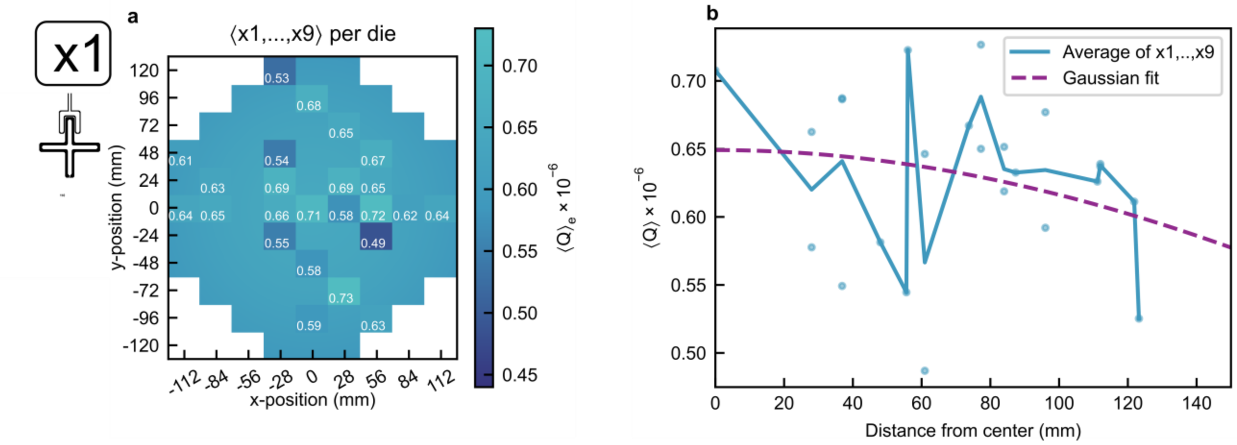

SUPPLEMENTARY FIG. 8 **Sub-die D2 Q-factor wafer map.** **a**, Average qubit quality factor $Q = 2\pi f_{qb}\langle T_1\rangle_t$, averaged over all fixed frequency qubits (x1,…,x9 in Supplementary Table I) with the identical capacitor geometries on sub-die D2 across the wafer. The background colour represents the Gaussian fit to the average value as a function of distance from centre. **b**, Q-factors plotted as function of distance from the centre of the wafer, the solid line corresponds to the average value, while the dashed line is a gaussian fit, acting as a guide to the eye and used as the background colour in **a**.

1. Verjauw, J. *et al.* Path toward manufacturable superconducting qubits with relaxation times exceeding 0.1 ms. *Npj Quantum Inf.* **8**, 1–7 (2022).

2. Jeurgens, L. P. H., Sloof, W. G., Tichelaar, F. D. & Mittemeijer, E. J. Growth kinetics and mechanisms of aluminum-oxide films formed by thermal oxidation of aluminum. *J. Appl. Phys.* **92**, 1649–1656 (2002).

3. Martinis, J. M. *et al.* Decoherence in Josephson Qubits from Dielectric Loss. *Phys. Rev. Lett.* **95**, 210503 (2005).

4. Osman, A. *et al.* Mitigation of frequency collisions in superconducting quantum processors. *Phys. Rev. Res.* **5**, 043001 (2023).
